# Modeling the combined effects of changing land cover, climate, and atmospheric deposition on nitrogen transport in the Neuse River Basin^☆^

**DOI:** 10.1016/j.ejrh.2018.05.004

**Published:** 2018-08

**Authors:** Mark Gabriel, Christopher Knightes, Ellen Cooter, Robin Dennis

**Affiliations:** a USEPA/Office of Research and Development(ORD), National Exposure Research Laboratory (NERL), Ecosystem Research Division (ERD), 960 College Station Rd., Athens, GA, 30605, USA; b USEPA/ORD/NERL/Atmospheric Modeling and Analysis Division (AMAD), 109 T W Alexander Drive, Research Triangle Park, NC, 27711, USA

**Keywords:** GCM, Climate change, Nitrogen, Land cover, CO_2_, Clean Air Act

## Abstract

**Study region:**

The SWAT model was used to estimate the combined effects of changing land cover, climate and Clean Air Act (CAAA)-related atmospheric nitrogen (N) deposition to watershed nitrogen fate and transport for two watersheds in North Carolina, USA.

**Study focus:**

Two different model simulation scenarios were applied: one included CAAA-related atmospheric N deposition, climate and land cover (CAAD+C+L) and the other only included CAAA-related N deposition (CAAD) in simulation.

**New hydrological insights for the region:**

Results show both scenarios generated overall decreasing trends for nearly all N outputs between 2010 and 2070 which resulted primarily from CAAA-related reductions in oxidized N deposition. In both watersheds, including climate and land cover change in simulation resulted in a relative 30% higher NO3 load, 30% higher denitrification, 10% higher organic N load and a 20% smaller level of plant N uptake in year 2070 compared to not including climate and landcover changes in simulation. The increases in N transport for both watersheds indicates the combined impacts from climate and land cover change may offset benefits provided by the CAAA regulations; however, future NO_3_ loads for the Little River watershed were small relative to current N loading rates. Conversely, the increasing NO_3_ and organic N loads for the nearby Nahunta watershed were significant compared to current rates demonstrating that watershed nutrient responses to climate and land cover changes may vary significantly over relatively small spatial scales.

## 1. Introduction

Global climate change is expected to present significant changes to seasonal and long-term variability of surface flows, groundwater flows and water quality. In particular, a greater occurrence of extreme meteorological events is anticipated (Cambell et al., 2009; Ficklin et al., 2010; Johnson et al., 2012; Van Liew et al., 2012; Dayyani et al., 2012, Sellamia et al., 2016). With a steady increase in global population, urban development quickly follows, which also heavily influences watershed hydrology and pollutant load delivery (Ferrier et al., 1995; Sobota et al., 2009; Wilson and Weng 2011; Wiley et al., 2010; Tang et al., 2011; Bosch et al., 2014). Watershed systems are highly sensitive to climate conditions. In many areas, climate change is expected to exacerbate current stresses on water resources from population and economic growth, land use change and urbanization (Butcher et al., 2013). A recent report by a committee of business and policy leaders say the US economy could face significant disruptions from climate change. Of particular concern are impacts to ecosystem function and expansion of anoxic regions in oceans that is further confounded by uncertainties inprojected climate change (Showstack, 2014).

Currently, there is a relatively large literature base concerning impacts of climate and land cover change on watershed hydrology and water supply, however water quality and upland biogeochemical responses have been studied much less in this context (Tu, 2009; Han et al., 2009, Kundzewicz, 2010; Park et al., 2010, 2011; Dayyani et al., 2012; Chiang et al., 2012; Johnson et al., 2012; Van Liew et al., 2012; Whitehead and Crossman, 2012, Bussi et al., 2016). Furthermore, few studies have analyzed the combined effects of land cover and climate change on nutrient transport (Bierwagen et al., 2010; Park et al., 2011; Van Liew et al., 2012; Astaraie-Imani et al., 2012, Johnson et al., 2015, Bussi et al., 2016). Water quality responses to changes in climate are difficult to predict because of complex biogeochemical cycling in aquatic and upland environments (Howarth et al., 2006; Cambell et al., 2009; Bernal et al., 2012). Issues regarding nitrogen transport under climate change involve not only changes in short term delivery but also transformations in landscape nitrogen sinks (storage in soils and biomass or rates of denitrification) (Aber et al., 2002; Howarth et al., 2006). Elevated levels of nitrogen in freshwater systems, estuaries and coastal areas are of concern due to nitrogen’s role in water-quality degradation, eutrophication and hypoxia (Smith et al., 1999; Galloway et al., 2004; Compton et al., 2011; Passeport et al., 2012).

Along with climate and land cover, the characteristics of atmospheric nitrogen deposition have a major impact on nitrogen transformation and delivery in watersheds. Atmospheric pollutant composition and concentration is heavily impacted by the type and intensity of industrial emissions. In the US, emissions regulation is enforced under the US Environmental Protection Agency’s (USEPA) Clean Air Act (CAA) (1963, 1967, and 1970) and the Clean Air Act Amendments (CAAA) of 1977 and 1990 (U.S. Environmental Protection Agency (USEPA), 1999; Butler et al., 2005). A primary goal of CAAA is to reduce ecosystem damage associated with low pH (acid) deposition in the eastern US and eastern Canada (Butler et al., 2001).

Investigations combining climate, land cover and atmospheric nitrogen deposition change into one modeling framework to evaluate long term projections in ecosystem nutrient dynamics are limited in number and scope (Civerolo et al., 2008; Cambell et al., 2009, Pan et al., 2009; Shi et al., 2011, Bussi et al., 2016), largely because of difficulties in linking various data sources and biogeochemical modeling components. Climate, land cover/use and atmospheric deposition represent primary factors affecting global water quality and nutrient balance (Williamson et al., 2008; Park et al., 2010; Churkina et al., 2010) therefore modeling investigations including these factors could provide a comprehensive evaluation of the broad spectrum of global influence which can lead to more accurate predictions of water quality and better management of natural resources.

A previous study by this research group showed that the decrease in CAAA-related atmospheric nitrogen deposition from 1990 to 2010 over the Neuse River Basin region correlates with a decrease in nitrogen discharges from the Little River and Nahunta watersheds (Gabriel et al., 2014b). In a separate climate and land cover change investigation, Gabriel et al., 2016 separately tested the influence of climate and land cover changes to determine the relative sensitivity of climate and land cover on nitrogen discharges for years 2010–2070. They showed nitrogen watershed discharges increase with increasing ambient CO_2_, decrease with land cover urbanization and have a mixed response to precipitation and ambient temperature fluctuations. This study also showed nitrogen watershed discharges were much more sensitive to the combined effects of precipitation and temperature than CO_2_ and land cover changes.

The purpose of the study presented here is to further build this recent work by Gabriel et al. by combining climate, land cover and CAAA-related changes in atmospheric nitrogen deposition into one modeling framework to reveal the combined influence of all three on nitrogen fate and transport for these in these two watersheds (Little River and Nahunta in North Carolina, USA). We also ran a series of simulations that do not contain climate and land cover changes; only CAAA-related changes in atmospheric deposition were included. This was completed to determine the relative influence of climate and land cover changes on the system, because over the long term, the benefits of CAAA regulations on nitrogen discharges may be offset or further enhanced with the advancement of climate and land cover changes (Civerolo et al., 2008).

For this study, we once again used the Soil and Water Assessment Tool (SWAT) watershed model for all watershed simulations and extracted output data for nitrogen (NO_3_ and organic nitrogen) stream/river discharge, upland denitrification and plant nitrogen uptake. We chose nitrogen discharge, denitrification and plant nitrogen uptake as the response variables because each are primary pathways for watershed nitrogen removal. Nitrogen discharges are a final product of the interaction between upland biogeochemistry, atmosphere-surface exchange, hydrology, land cover change, land management practices and are the focus of many pollution abatement programs, e.g. Total Maximum Daily Loads. Denitrification is a difficult process to experimentally measure as it occurs in small anaerobic pockets in soil and depends on NO_3_ availability, carbon availability, temperature and substrate composition (Donner et al., 2004) and can vary dramatically with climate variation (Groffman et al., 2009); therefore, model simulations that provide estimates of denitrification including plant uptake are particularly valuable. The climate data used in this study involves ambient CO_2_, precipitation and temperature. CO_2_ data were obtained from estimates determined by the International Panel on Climate Change (IPCC) and future estimates for precipitation and temperature were obtained from two statistically downscaled Global Circulation Models (GCMs). Land cover change predictions were obtained from the US Environmental Protection Agencies (USEPA) Integrated Climate and Land Use Change (ICLUS) project and the USEPA’s Community Multi-scale Air Quality (CMAQ) model was used to obtain future estimates for atmospheric nitrogen deposition.

The study presented here is essentially a single-scope scenario analysis since we consider one atmospheric deposition projection scenario beyond 2010, one climate and one land use (land cover) change scenario (A2; “business as usual” scenario); therefore, the results presented are one of many possible future outcomes. However, we do examine two extreme climate scenarios (wet-cold and dry-warm) including the predominant projected land cover conversion (agricultural and forested to urban) for the studied region. The novelty of this study partly lies in the exercise of linking three complex datasets into a SWAT model framework; climate, land cover and CAAA-related atmospheric deposition. We rely on the findings discussed in Gabriel et al. (2014a, 2014b, 2016) to dissect and analyze the modeling results presented in this manuscript. Part of the motivation for developing these past studies was to build a knowledge base and an appropriate modeling platform in order to evaluate the combined effects from changing climate, land cover and atmospheric nitrogen deposition in SWAT simulation for the study watersheds presented in this manuscript.

## 2. Study area description

We performed this modeling investigation in two hydrologic unit code (HUC) 10 watersheds located within the Neuse Watershed of North Carolina, USA: the Little River and the Nahunta. The Little River watershed drains an area of 202.5 km^2^ at USGS gauging station 208521324 (Little River at SR1461 near Orange Factory; NC; 36.142 [Lat.], −78.919 [Long.]). The Nahunta watershed drains an area of 207.2 km^2^ at USGS flow gauging station 2091000 (Nahunta Swamp near Shine, NC; 35.489 [Lat.], —77.806 [Long.]). Little River is located in the Piedmont region and Nahunta is in the Atlantic Coastal Plain. These watersheds were selected because of contrasting land cover characteristics, location in the Neuse and availability of observed data (flow and nitrate) for calibration purposes. Physiographic information for each watershed is provided in Table 1. See Gabriel et al. (2014a) for more details on the study watersheds.

## 3. Model, data and methods

### 3.1. Model application

The SWAT watershed model was used for this investigation. A revised version of the SWAT 2009 code (477 dated 4/15/13) was developed for this study to allow entry and computation of spatio-temporal varying (on a sub-basin and monthly basis) atmospheric nitrogen deposition and ambient CO_2_. SWAT was chosen for this modeling framework because of its wide user base for hydrologic and nutrient simulations and its appropriate application for the selected watersheds which are largely agricultural and forest-based. For information on the simulation of nitrogen cycles in SWAT, data input and processing and model sensitivity analysis refer to the Supporting Information section.

### 3.2. Land cover and climate change data

#### 3.2.1. Land cover

The land cover change data was obtained from USEPA’s Integrated Climate and Land Use Change Research Program (ICLUS) (http://www.epa.gov/ncea/global/iclus/). ICLUS produces national-scale change scenarios for urban and residential development underlying different IPCC greenhouse gas emission storylines. The scenarios use a demographics model to estimate population through 2100 for the conterminous US, which is then allocated to 1 ha pixels. The final spatial dataset provides projections for housing density and impervious surface cover every five years, from 2000 through 2100 (Bierwagen et al., 2010; Johnson et al., 2012). To be consistent with CO_2_ projections, we considered a single future land cover (land use) change scenario representative of the IPCC A2 greenhouse emission storyline. ICLUS data were separately obtained for both watersheds. ICLUS projects these watersheds will undergo substantial conversion to urban cover, according to the A2 storyline. By 2030, ICLUS projects 69% of the Little River watershed and 22% of the Nahunta watershed will have converted to urban cover. To incorporate projected land cover changes, we modified the reference land cover areas provided by the NLCD files, relative to ICLUS projections (see Gabriel et al., 2016 for more information).

#### 3.2.2. CO_2_

Future projections for ambient CO_2_ were obtained from estimates provided by the Intergovernmental Panel on Climate Change (IPCC) (Intergovernmental Panel on Climate Change (IPCC), 2007) (Gabriel et al., 2016). Ambient CO2, an important driver of climate change, was included in the assessment because of its direct impacts to water balance and vegetation growth in the SWAT model, as in any natural system. The projected CO_2_ levels used here are representative of the A2 greenhouse gas storyline which assumes a heterogeneous world of self-reliance and preservation of local identity, high energy use; medium-to-high rates of land cover change and fertility patterns across regions that converge very slowly, resulting in a continuously increasing global population. The A2 storyline is commonly referred to as a “business as usual” case since it reflects socio-economic and industrial emissions conditions comparable to the current time period (Intergovernmental Panel on Climate Change (IPCC), 2007). The simulated response of water quality/quantity to climate and land use change depends heavily on the IPCC storyline used in simulation (Wilson and Weng, 2011); therefore, results for this research are only relevant to future conditions that reflect the A2 storyline. For the reference CO_2_ condition, a constant value of 380 ppm (current conditions) was used which represents current global conditions. In this study, potential evapotranspiration (PET) was determined using the Penman-Monteith equation where CO_2_ directly affects PET and, subsequently, ET through modification of leaf stomatal conductance and the leaf area index (LAI). ET is a primary determinant for surface runoff in SWAT (Neitsch et al., 2005).

#### 3.2.3. Precipitation and ambient temperature

Model projections for precipitation and ambient temperature were based on bias-corrected and statistically downscaled data from the ECHO (Hamburg Atmosphere-Ocean Coupled Circulation model) and CCSM3 (Community Climate System model) models. These data were obtained from the 12 km CONUS Daily Downscaled Climate Projections developed by Katherine Hayhoe and others at the U.S. Geological Survey; see USGS Data Portal at www.cida.usgs.gov/climate/gdp. We chose the ECHO and CCSM3 CGMs because they present climactic contrasts for the North and South Carolina region (Conrads et al., 2012). ECHO predicts drier, warmer conditions and CCSM3 predicts wetter, cooler conditions (see Gabriel et al., 2016). Similarly to the CO_2_ and land cover criteria, ECHO and CCSM3 data predictions are representative of the A2 greenhouse emission storyline. See Section 3.2.4 for a description on how reference precipitation and temperature data were developed. Even though ambient temperature has a major impact on all ecohydrologic processes, namely, evapotranspiration, chemical reaction kinetics and microbial-mediated processes (Whitehead and Crossman, 2012), precipitation and temperature were combined as one treatment because, in a previous study by these authors, ambient temperature had minor impacts on nitrogen discharge compared to precipitation for these watersheds (Gabriel et al., 2014a).

#### 3.2.4. Reference data development for precipitation and temperature

Reference data was developed for precipitation and temperature as was done for CO_2_ and land cover. The reference precipitation and temperature data sets were developed by de-trending (creating zero slopes) GCM data. For GCM precipitation, there were no long-term daily trends, but there were trends for individual months over the 60-year period, e.g., all Januaries from 2005 to 2070 (see Section 3.2.3). Reference precipitation data was developed by subtracting the product of monthly slopes and number of years from the daily data (Eq. (1)); this de-trended increasing and decreasing monthly slopes. For daily temperature (Eq. (2)), both data sets showed statistically significantly (p < 0.001) positive increases and were de-trended to create zero slopes. This was done for both daily minimum and maximum temperature data. GCM in these equations refers to ECHO and CCSM3 since we developed separate reference data for both models.

1 $${\text{ReferencePrecip}}_{\text{yeari,dayi}}{\text{ = GCM}}_{\text{yeari,dayi}}{\text{ - (GCM}}_{\text{slope,month}}\text{ * years)}$$

years=years since 2005

slope=slope of daily totals for the same month from 2005 to 2070

2 $${\text{ReferenceTemp}}_{\text{yeari,dayi}}{\text{ = GCM}}_{\text{yeari,dayi}}{\text{ - (GCM}}_{\text{slope}}\text{ * days)}$$

days=days since 1/1/2005

slope=slope of the GCM trend line from 2005 to 2070

### 3.3. Atmospheric nitrogen deposition

The USEPA’s Community Multi-scale Air Quality (CMAQ) Version 4.6 modeling system was used to obtain monthly atmospheric nitrogen deposition data (Byun and Schere, 2006; U.S. Environmental Protection Agency (USEPA), 2012a, 2012b). CMAQ is a publicly available, peer reviewed, state-of-the-science model that simulates multiple chemical and physical processes important to understanding atmospheric trace gas transformations and distributions. The atmospheric nitrogen deposition data obtained from CMAQ reflect expected emissions measures implemented since 1990 to comply with rules promulgated through September 2005 while allowing for changes in population and economic activity including emissions attributable to economic and population growth (U.S. Environmental Protection Agency (USEPA), 2011). The following CAAA program controls are modeled in CMAQ: (1) Title I VOC and NOx reasonably available control technology requirements in ozone nonattainment areas; (2) Title II on-road vehicle and nonroad engine/vehicle provisions; (3) Title III National Emission Standards for Hazardous Air Pollutants; (4) Title IV acid rain programs focused on emissions from EGUs; and (5) additional EGU regulations, such as the Clean Air Interstate Rule, the Clean Air Mercury Rule, and the Clean Air Visibility Rule. Types of emission sources considered were EGUs (electricity production), non-EGUs (e.g. industrial boilers, cement kilns), on-road motor vehicles (e.g. buses, cars, trucks), non-road engines/vehicles (e.g. aircraft, construction and lawn/garden equipment), and area sources (e.g., dry cleaners, wildfires). Climate or land cover changes were not considered in CMAQ but we incorporate these using the ICLUS, IPCC and GCM data as previously described. For discussion on model QAQC, uncertainties and assumptions for CMAQ data, refer to Benefits and Costs of the Clean Air Act from 1990 to 2020 (U.S. Environmental Protection Agency (USEPA), 2011). At the time of this study CMAQ simulations were generated for 36 km or 12 km grid sizes. The 36 km grid data was used for this study. The CMAQ nitrogen deposition data used in this study was obtained from a collaborating agency in support of generating results for this report.

Table 2 shows annual summaries of the CMAQ data. All trends from 2000 to 2020 increased at various rates except for oxidized nitrogen. These trends are clear since CAAA only targets oxidized nitrogen and does not regulate NH_3_ emissions, which are projected to increase (Dennis et al., 2010) due to growing demand for food (Paulot et al., 2013). Reduced nitrogen is larger for the Nahunta watershed because of a relatively high concentration of confined animal feeding operations (CAFO) in the Nahunta watershed regional area. NH_3_ emissions are high in cases of fertilizer application and animal feeding operations (Civerolo et al., 2010). Wet deposition concentration data agrees well with National Atmospheric Deposition Program data up through 2010 (http://nadp.sws.uiuc.edu;. stations NC41, NC35) (Gabriel et al., 2014a). There were significant contrasts (in magnitude and trends) between CMAQ and CASTNet (another commonly used US dry/wet deposition data set) wet and dry fluxes because (1) CASTNet data are estimates using a different multi-layer dry deposition model (2) CMAQ uses a repeated precipitation year (2002) in simulations and (3) CASTNet does not include many nitrogen species, e.g. NH_3_, PAN, NO_2_ and NO, that are included in CMAQ simulation.

**Fig. 1.**
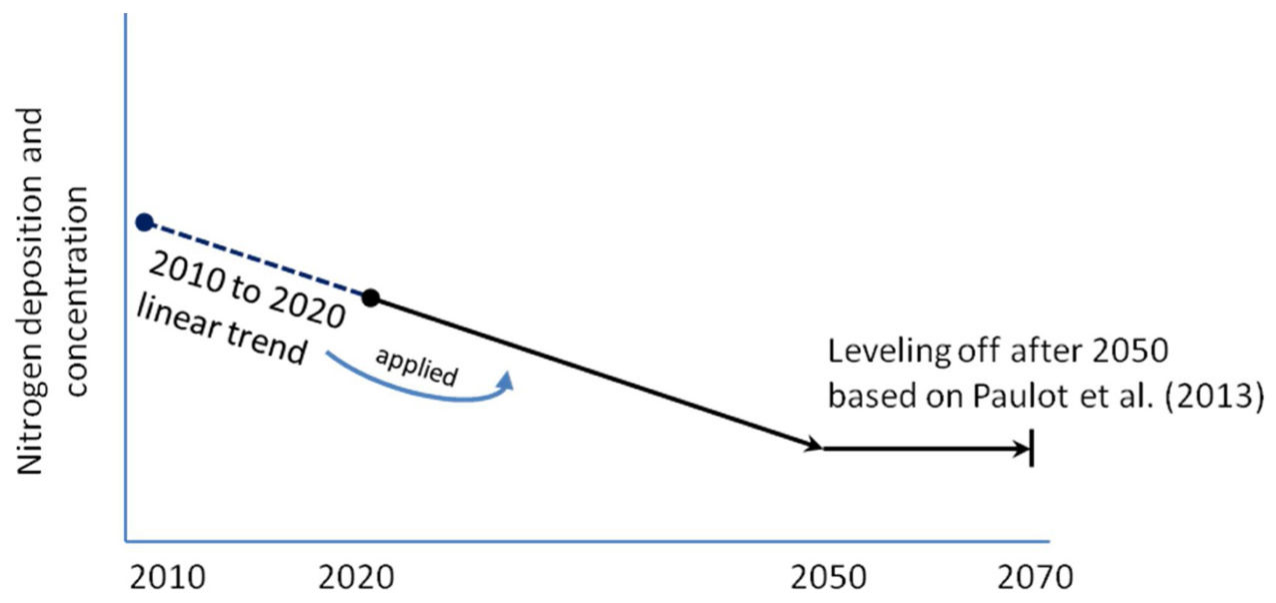
A schematic showing the method used to develop monthly nitrogen deposition and concentration data from 2020 to 2070 for both increasing and decreasing trends: CMAQ trends are from 2010 to 2020. We extended the 2010–2020 linear trends out to 2050. After 2050, we flattened all trends. These trend extension and de-trending techniques reflect the modeled trends for NH_x_ and NO_y_ species determined by Paulot et al. (2013).

**Fig. 2.**
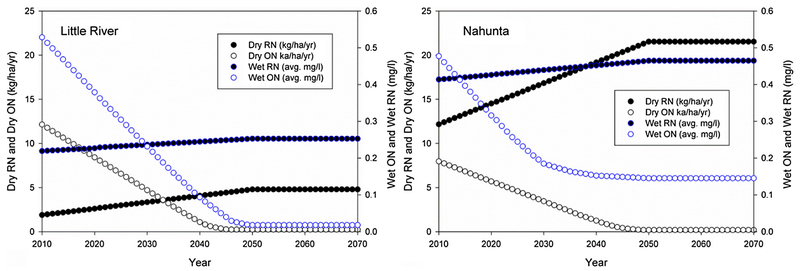
Trends for dry and wet nitrogen deposition and concentrations in both watersheds: Data from 2010 to 2020 were generated from CMAQ. Data beyond 2020 was developed using the methods described in Section 3.3.

To develop monthly deposition and concentration data beyond year 2020 we first developed linear trends for each nitrogen specie between years 2010 and 2020, then applied these trends to generate data up to year 2050 (see Figs. 1 and 2). Extending these trends to 2050 and zero-trending thereafter is similar to modeled data for NH_x_ (NH_3_ + NH_4_^+^) and NO_y_ (NO_x_ + NO_3_ + 2N_2_O_5_ + HONO + HO_2_NO_2_ + organic nitrates; NO_x_ = NO + NO_2_) determined by Paulot et al. (2013). Paulot et al. (2013) provide a broad assessment of projected deposition for various reactive nitrogen species in two US National Parks, among other locations using the GEOS-Chem global chemical transport model and current air regulation information. In this Paulot study, trends for NH_x_ and NO_y_ between 2000 and 2010 are similar to the CMAQ data in this study; however, there are contrasts in nitrogen levels because of differing study locations. As shown in Fig. 2, oxidized nitrogen trends curve as the data approaches year 2050 because of averaging over several years that contain high monthly variability.

## 4. Results and discussion

### 4.1. Trends and spatiotemporal variations in nitrogen outputs

**Fig. 3.**
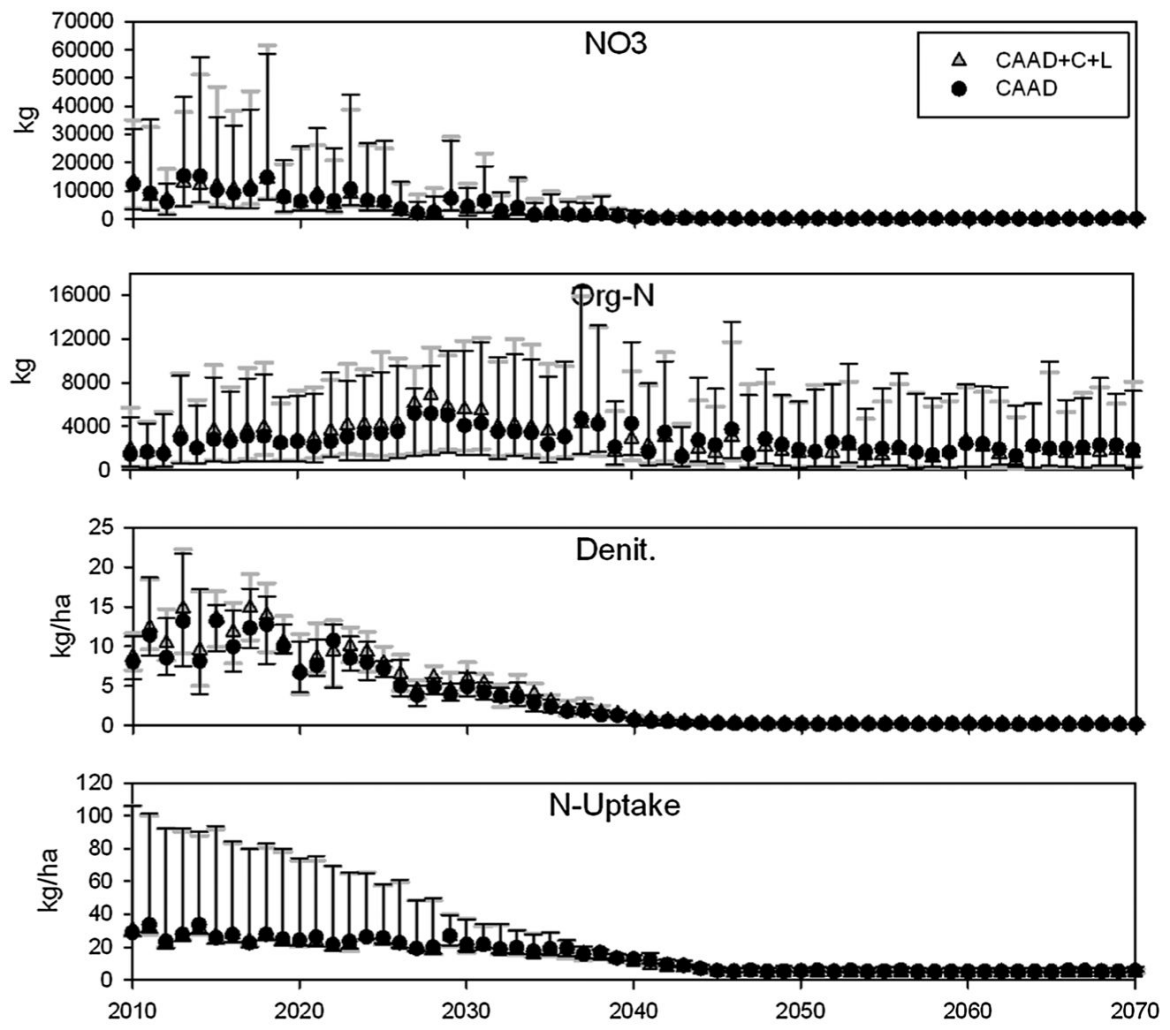
SWAT simulation results for nitrate (NO_3_), organic nitrogen (Org-N), denitrification (Denit) and plant nitrogen uptake (N-uptake) for the Little River watershed: The CAAD+C+L scenario refers to CAAA-related changes in atmospheric nitrogen deposition, climate and land cover change in SWAT simulation. The CAAD scenario only includes CAAA-related changes in atmospheric nitrogen deposition in simulation. Climate change includes CO_2_, precipitation and temperature. For each year results show the median, 25th and 75th percentiles for all sub-basins (only for NO_3_ and Org-N) and HRUs (only for Denit and N-uptake).

**Fig. 4.**
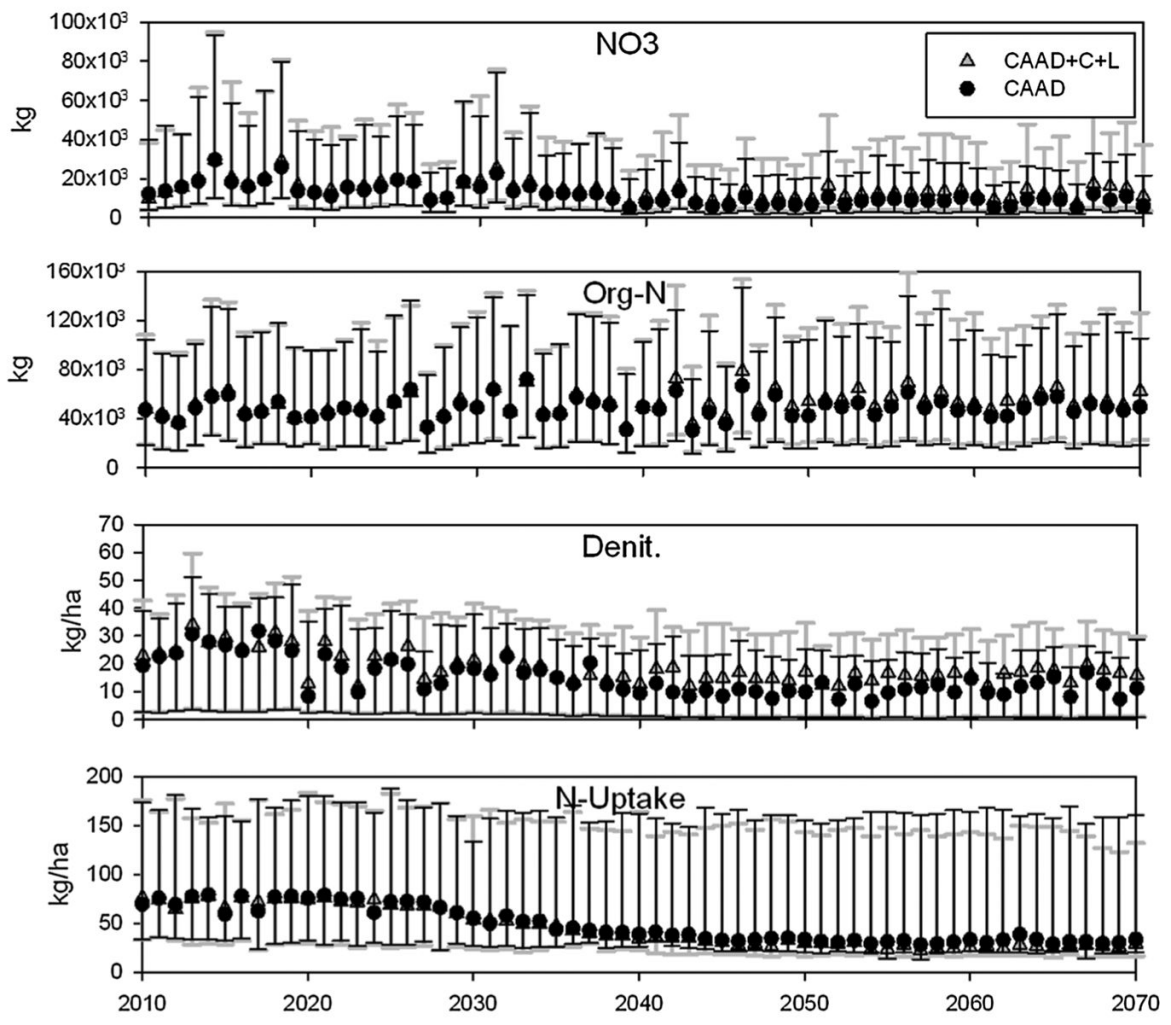
SWAT simulation results for nitrate (NO_3_), organic nitrogen (Org-N), denitrification (Denit) and plant nitrogen uptake (N-uptake) for the Nahunta watershed: The CAAD+C+L scenario refers to CAAA-related changes in atmospheric nitrogen deposition, climate and land cover change in SWAT simulation. The CAAD scenario only includes CAAA-related changes in atmospheric nitrogen deposition in simulation. Climate change includes CO_2_, precipitation and temperature. For each year results show the median, 25th and 75th percentiles for all sub-basins (only for NO_3_ and Org-N) and HRUs (only for Denit and N-uptake).

Figs. 3 and 4 show SWAT model simulation results for upland denitrification (Denit), plant nitrogen uptake (N-uptake), sub-basin nitrate (NO_3_) and organic nitrogen (Org-N) loading. There are two different simulation scenarios. The first scenario included CAAA-related changes in atmospheric nitrogen deposition, climate and land cover in SWAT simulation (CAAD+C+L) and the second scenario only included CAAA-related changes in nitrogen deposition (CAAD). Reference data (see Section 3.2.4) was used in place of the climate and land cover change data for the CAAD scenario. A visible feature for nearly all plots in Figs. 3 and 4 are the decreasing trends for NO_3_, Org-N, Denit and N-uptake in both watersheds. The cause for the decreasing trends in both scenarios is largely decreasing atmospheric nitrogen deposition (Fig. 2), because decreasing trends are present even with de-trended precipitation (P), ambient temperature (T), CO_2_ and constant land cover percentages in the CAAD scenario. The strong influence of atmospheric deposition is reinforced in Tables 3 and 4 (correlations were completed for the CAAD+C+L scenario only). Gabriel et al. (2014b) found that the oxidized component of atmospheric nitrogen had the largest influence on nitrogen discharges. This is also evident in this study since oxidized nitrogen is the only specie that decreases over time (Fig. 2). Org-N discharge in both watersheds shows the most irregular/unique trends. For Nahunta, there is a zero-slope trend and a unimodal trend for Little River, with the mode around year 2027 (Fig. 3). This zero-slope trend for Nahunta is surprising since atmospheric nitrogen deposition, P, T do not have a zero-slope trend (see Section 3.2.3). However, in Gabriel et al. (2016) when precipitation and temperature (PT) act alone on Org-N, it causes an increase in Org-N loading and atmospheric nitrogen deposition causes a decrease in Org-N (this study). Their combined effect in simulation likely causes the zero-slope trend. The unimodal trend for Org-N in Little River is consistent with a drop in PT around year 2027 (Gabriel et al., 2016) further reinforcing the large, combined influence of P and atmospheric nitrogen deposition on nitrogen discharges. The decreasing trend for NO_3_ (particularly for Little River) in the CAAD+C+L scenario is encouraging (Figs. 3 and 4) and indicates that, when combined with CAAA regulations, climate and land cover changes did not cause an increase in NO_3_ loading over time; however, that is not the case in a relative mass loading context which will be scrutinized in the next section.

The inter-quartile ranges for all outputs in Nahunta are much greater than Little River which is connected to greater variety of soil classes and land cover types which generates greater variability in nitrogen fate and transport results (Figs. 3 and 4). The magnitudes for Nahunta output values are more than twice as large for N-uptake and Denit and an order of magnitude greater for NO_3_ and Org-N which is due to greater fertilizer applications (manure and pure nitrogen [agricultural lands] and urea [urban lands]) over the simulation period for Nahunta (avg. 45.0 kg/ha/yr) than Little River (avg. 1.5 kg/ha/yr).We parameterized fertilizer applications in SWAT using a fixed schedule and amount for each crop each year. This was done to provide more controlled conditions so as to clearly reveal the combined impacts of climate change, land cover change, and CAAA-related changes in N deposition. For each year, the highly skewed data distributions for N-uptake in Little River are due to hay land cover which, on average, took up an order of magnitude more nitrogen than all other land covers and has a large surface area (Table 1). For Nahunta, the distributions are skewed toward the later years because N-uptake is higher for soybean, corn, cotton, hay and spring wheat. These land covers have the largest surface areas for this watershed and decreased in very little in coverage over time with projected land cover changes. See Gabriel et al. (2014b) for a detailed presentation and discussion of how N transport (NO_3_, Org-N, N-uptake, Denit) differs as a function of multiple land cover types in both watersheds.

### 4.2. Relative influence of climate and land cover change on nitrogen transport

**Fig. 5.**
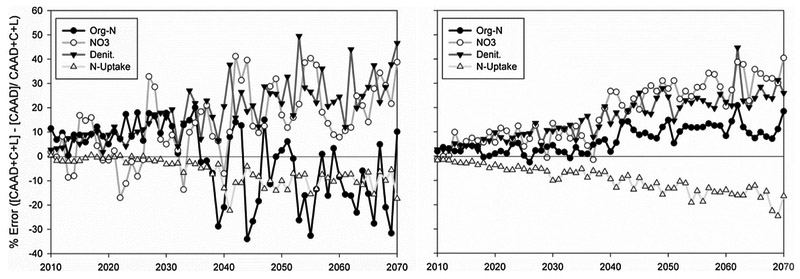
Little River (left) and Nahunta (right): Results show median values over all HRUs (Denit, N-uptake) and sub-basins (Org-N, NO_3_). The CAAD+C+L scenario refers to CAAA-related changes in atmospheric nitrogen deposition, climate and land cover change in SWAT simulation. The CAAD scenario only includes CAAA-related changes in atmospheric nitrogen deposition in simulation. Percent errors were calculated with data used in Figs. 3 and 4. Percent error values above zero indicate higher Org-N, Denit, N-uptake and/or NO_3_ levels with climate and land cover change. Vice-versa for values below zero.

In Fig. 5 there are visible differences in results between the CAAD+C+L and CAAD scenarios indicating that, under this modeling set-up, the combined effects of climate and land cover changes will generate gradual increases in NO_3_ loading and Denit, a decrease N-uptake and have a mixed response for Org-N loading. Percent errors (differences) were calculated with data used in Figs. 3 and 4. Percent error values above zero indicate higher Org-N, Denit, N-uptake and/or NO3 levels with climate and land cover change. Vice-versa for values below zero. The steady increase in percent changes indicate that climate and land cover change become important over time to nitrogen transport as atmospheric nitrogen deposition decreases as a result of CAAA regulations (Fig. 5). It is also important to note that while NO_3_ loading is approximately 30% greater in year 2070 under the CAAD+C+L scenario (climate and land cover changes factored in) for both watersheds, the loads are minimal compared to present NO_3_ loading rates for Little River. In contrast, for Nahunta the advancement of climate and land cover change (CAAD+C+L scenario) may generate more noticeable increases in NO_3_ and Org-N loading (Figs. 3 and 4).

The sensitivity analysis conducted by Gabriel et al. (2016) can help determine which forcing function/parameter(s) (P. T, CO_2_, etc.) is most responsible for the change in nitrogen transport under the CAAD+C+L scenario. Gabriel et al. (2016) showed NO_3_ and Org-N loadings increase with increasing CO_2_, have an overall decrease with land cover change (conversion from agricultural and forests lands to urban) and a mixed response for PT changes (increase in loading for Nahunta and no trend for Little River). In addition, NO_3_ and Org-N had ~10x more sensitivity to PT changes than land cover and CO_2_ change and land cover and CO_2_ had roughly equal levels of influence on nitrogen discharges. In this study, the only parameters that could cause the observed increase in NO_3_ loading in both watersheds are CO_2_, P and T because urbanization causes a decrease in NO_3_ loading for these watersheds. CO_2_ can increase nitrogen loading through changes in plant stomatal conductance. SWAT models CO_2_ effects on plant growth based on research by Morrison (1987) and Easterling et al. (1992) who found that increased atmospheric CO_2_ lowers stomatal conductance because plants transpire a smaller amount of water to obtain the CO_2_ they need for growth, thereby reducing transpiration and overall evapotranspiration (ET). A reduction in ET creates a shift in upland water balance toward increased soil water content and plant water efficiency, leading to increased runoff and increased NO_3_ loading (Betts et al., 2007; Wu et al., 2012). As mentioned, we found CO_2_ had minimal impacts in nitrogen loading compared to PT. Even though an increase in CO_2_ alone causes an increase in nitrogen loading (also seen by Wu et al., 2012 and Butcher et al., 2014), the inverse correlation between CO_2_ and NO_3_ (Tables 3 and 4) is expected because of the overwhelming influence of atmospheric nitrogen deposition. In SWAT there are no atmospheric-related interactions between ambient CO_2_, P and T therefore this can be ruled out as a secondary influence. Lastly, a simple comparison of the profiles for NO_3_ percent increase (Fig. 5) and NO_3_ loading under the PT sensitivity analysis (Gabriel et al., 2016) reveals stark similarity. Therefore, we estimate that the primary cause for the steady increase in NO_3_ for the CAAA+C+L scenario is from combined PT changes; mainly P, however since Gabriel et al. (2014a) showed T had minimal impacts on nitrogen fate and transport compared to P for these watersheds. The changes in percent error for Org-N in both watersheds are also primarily from P for the same reasons as NO_3_. This is evident by observing the similar profiles between percent changes and absolute Org-N loads acting under PT (Gabriel et al., 2016).

Denit and N-uptake also show clearly visible percent error differences between both scenarios (Fig. 5). In SWAT, relevant model terms/variables that increase Denit are soil nitrate concentration, moisture and temperature. N-uptake is a product of plant biomass production, soil nitrogen and heat unit regulation (see SWAT model theoretical documentation at http://swat.tamu.edu/ documentation/for details); the latter two terms are a functions of precipitation, temperature and atmospheric nitrogen deposition. Plant biomass production is a function of the plant type. Nitrogen plant uptake is calculated as the difference between the actual concentration of nitrogen in the plant and the optimal concentration. In the case of legumes (e.g. alfalfa), if the soil cannot meet the daily nitrogen demand, the deficit is attributed to nitrogen fixation (Varanou et al., 2002). NO_3_ uptake by plants increases with temperature (Pourmokhtarian et al., 2012). Since we previously did not conduct a sensitivity analysis on Denit and N-uptake (as was done for Org-N and NO_3_) it is challenging to pinpoint the exact parameter(s) that is most important to the change of Denit and N-uptake under the CAAA+C+L scenario. Denit however shows a very similar response to NO_3_ loading (Fig. 5). The increase in Denit over time was not from soil nitrogen levels, which decreased over time with a decrease in atmospheric oxidized nitrogen deposition. Therefore, the increase in Denit was from an increase in ambient temperature (see Section 3.2.3) and/or soil moisture. From the gradual increase in precipitation, soil moisture levels increased in both watersheds. For example, under the CAAA+C+L scenario (combining ECHO and CCSM3 GCM data) average soil water content levels in Little River rose 5.2% by year 2070. N-uptake is unique in that it shows a consistent decline in the CAAA+C+L scenario. Since P and T increase over time, the only parameters that could have caused the reduction in N-uptake are the rising CO_2_ and land cover conversion (urbanization). As mentioned, increasing CO_2_ decreases ET which creates a shift in upland water balance toward increased soil water content and plant water efficiency therefore requiring less nitrogen for growth, thereby decreasing N-uptake overtime. Adding to this, the reduction profile for N-uptake is more similar to CO_2_ than urbanization (Gabriel et al., 2016)) therefore it is likely CO_2_ was the parameter primarily responsible for the decrease in N-uptake for the CAAA+C+L scenario.

## 5. Implications for changes in watershed nitrogen discharge/loading under future climate, land cover and CAAA change

These modeling results show that the combined effects from climate and land cover change may offset the benefits CAAA regulations have on reducing NO_3_ and Org-N loadings to receiving waters within the Neuse River Basin. Both watersheds show a median 30% higher NO_3_ load in 2070 (Fig. 5) under climate and land cover change conditions as delivered by the A2 scenario. The relative impacts of climate and land cover change appear to be more dramatic for Nahunta, which is the more agricultural based watershed. In Nahunta, certain crop types show continued release of elevated NO_3_ because (1) atmospheric deposition of oxidized nitrogen decreased at a slower rate over Nahunta and (2) there was a greater percentage of crop types that took up and released higher nitrogen loads, specifically, soybean, corn, hay and cotton. Org-N also showed an appreciable load increase (10–15% higher) in 2070 for Nahunta however loading was much more variable in Little River due to a shift in precipitation impacts around year 2027 (Gabriel et al., 2016).

Increases in nitrogen loading with climate and land cover change over time could have substantial implications for primary production and ecosystem services in the Neuse River Basin and Estuary. Eutrophication has been a water quality problem in the Neuse Estuary since post-World War II due to agricultural activities, urbanization and frequent hurricanes (Paerl et al., 2006a). This basin has a longstanding history of stress from hypoxia conditions (Fear et al., 2004; U.S. Environmental Protection Agency (USEPA), 2012a) and any additional increases in loading could pose deleterious impacts to fisheries and dissolved oxygen levels (Paerl et al., 2006a,b). As previously pointed out, while a 30% increase in nitrogen loading to receiving waters could be a major issue for Nahunta, the 30% increase for Little River may not because the loads by 2070 are only a fraction (e.g. one-tenth) of current loading levels. As such, the severity of projected climate and land cover impacts may be highly variable on a watershed basis within the Neuse River Basin and climate and land cover changes may have minimal impacts on nitrogen delivery assuming air regulations remain in place. These climate change impact results are unique. Very commonly, watershed modeling investigations indicate changing climate will generate negative, even cataclysmic environmental conditions over time. This study’s modeling results for the Little River watershed contrast that by showing changing climate will increase nitrogen loads however on a potentially trivial or negligible level. The key to further reductions in nitrogen loading is to ensure CAAA regulations do not erode or lessen over time.

Our statistical and modeling scenario analyses show that the combination of atmospheric nitrogen deposition and precipitation have the largest influence on nitrogen fate and transport in these watersheds. Changes in CO_2_ and land cover have a minor role. For land cover, this was largely because of the type of conversion that occurred (urbanization). Therefore, based on these modeling results, we suggest any future efforts made to improve and/or sustain ecosystem structure in the Neuse River Basin from a nitrogen management standpoint may be better achieved through investigation of current and/or planned air regulations and climate-related increases in precipitation. For example, valuable watershed nitrogen fate and transport investigations could include determining impacts of (1) various atmospheric chemistry and nitrogen deposition conditions that reflect with current and projected air regulations and (2) climate-related changes in precipitation patterns and frequencies.

## Supplementary Material

supp

## Figures and Tables

**Table 1:** Physical characteristics for the studied watersheds: The values shown were determined by the SWAT model.

Physical Characteristics		Watershed		
		Little River		Nahunta
Sub-basins		23		21
Surface Area (ha)		19734		16145
Min./Max. elevation (m)		109/244		16/70
NRCS Soil Classes		63		86
Hydrologic Response Units (HRU)		547		681

	SWAT Land Cover Categories		% Watershed Area	
			Little River	Nahunta

Agriculture	Corn (CORN)		1.44	6.36
	Upland Cotton (COTS)		-	6.16
	Grain Sorghum (GRSG)		-	0.03
	Soybean (SOYB)		1.64	22.53
	Peanut		-	1.60
	Tobacco (TOBC)		0.03	0.06
	Spring Barley (BARL)		0.01	-
	Winter Wheat (WWHT)		0.35	0.10
	Spring Wheat (SWHT)		0.62	9.19
	Rye (RYE)		0.06	-
	Oats (OATS)		0.01	0.01
	Pearl Millet (PMIL)		0.01	0.02
	Hay (HAY)		19.82	5.59
	Generic Agricultural Land (AGRL)		0.34	0.02
	Sweet potato (SPOT)		-	0.15
	Row Crop Agricultural Land (AGRR)		-	0.01
	Winter Pasture (WPAS)		0.48	0.37
	Tall Fescue (FESC)		4.67	2.81
	Range-Grasses (RNGE)		3.61	6.66
Urban	Low Density Residential (URLD)		4.91	5.52
	Medium Density Residential (URMD)		0.04	0.09
	High Density Residential (URHD)		0.01	0.02
	Water (WATR)		0.32	0.27
Forests	Mixed Forest (FRST)		5.16	1.61
	Deciduous Forest (FRSD)		47.25	9.70
	Evergreen Forest (FRSE)		8.22	7.47
Wetlands	Mixed Wetlands (WETL)		-	0.01
	Forested Wetlands (WETF)		0.99	13.53
	Non-Forested Wetlands (WETN)		-	0.09

**Table 2:** CMAQ atmospheric nitrogen concentration and deposition data for years 2000, 2010 and 2020 and data estimated by this study. These yearly summaries were developed from monthly data.

		Little River				Nahunta			
	Year	DryRN (kg/ha/yr)	DryON (kg/ha/yr)	WetRN (avg. mg/l)	WetON (avg. mg/l)	DryRN (kg/ha/yr)	DryON (kg/ha/yr)	WetRN (avg. mg/l)	WetON (avg. mg/l)
CMAQ	2000	1.30	22.1	0.21	0.89	10.4	13.4	0.41	0.76
	2010	1.88	12.1	0.21	0.51	12.3	7.76	0.41	0.47
	2020	2.60	8.42	0.22	0.36	14.7	5.49	0.42	0.31
Estimated by this study, see Fig. 1	2030	3.34	4.70	0.23	0.23	16.8	3.44	0.43	0.18
	:	:	:	:	:	:	:	:	:
	:	:	:	:	:	:	:	:	:
	2070	4.78	0.23	0.25	0.01	21.5	0.17	0.46	0.14

Compositions: Dry reduced nitrogen (DryRN): ANH4I, ANH4J, NH_3_, Dry oxidized nitrogen (DryON): ANO3I, ANO_3_J, NO_3_, N_2_O_5_, HONO, HNO_3_, NTR, PAN, PANX, Wet reduced nitrogen (WetRN): ANH_4_I, ANH_4_J, Wet reduced nitrogen (WetON): ANO_3_I, ANO_3_J, NO_3_, N_2_O_5_, HONO, HNO_3_, NTR, PAN, PANX,ANO_3_I – ultra-fine aerosol nitrate (0.01–0.1 μm), ANO^3^J – fine aerosol nitrate (0.1–1.0 μm), NO_3_-nitrate, N_2_O_5_–dinitrogen pentoxide, HONO – nitrous acid, HNO_3_–nitric acid, NTR – represents other organic nitrates to complete CMAQ mass balance in the chemical mechanism, PAN – peroxyacetylnitrate, PANX – higher order products of PAN, ANH_4_I – ultra-fine aerosol ammonium (0.01–0.1 μm),ANH_4_J – fine aerosol ammonium (0.1–1.0 μm), NH_3_– ammonia.

**Table 3:** Spearman rank correlations for Little River. Spearman rho (ρ) values are on top and *p*-values are below. Terms are color-coded to help with interpretation. Atmospheric deposition terms are in the yellow to red colors, temperature is in blue colors, precipitation is in green colors and CO_2_ is white. Land cover change was not included in this correlation analysis because there are only five urban change percentage values from 2010 to 2070 (see Gabriel et al., 2016).

	WETRN	WETON	DRYRN	DRYON	CO2	ECHOMINT	ECHOP	ECHOMAXT	CCSM3MINT	CCSM3MAXT	CCSM3P
Org-N	−0.608	0.608	−0.608	0.597	−0.587	−0.394	0.371	−0.325	−0.287	−0.149	0.135
	1.99E-07	1.99E-07	1.99E-07	4.16E-07	7.74E-07	1.76E-03	3.41E-03	1.08E-02	2.54E-02	2.49E-01	3.00E-01

	DRYON	WETRN	WETON	DRYRN	CO2	ECHOMINT	CCSM3MINT	ECHOMAXT	CCSM3MAXT	CCSM3P	ECHOP
NO_3_	0.944	−0.933	−0.933	−0.933	−0.9	−0.614	−0.603	−0.567	−0.402	−0.131	0.0118
	2.00E-07	2.00E-07	2.00E-07	2.00E-07	2.00E-07	1.30E-07	2.84E-07	2.26E-06	1.41E-03	3.15E-01	9.27E-01

	WETRN	WETON	DRYRN	DRYON	CO2	ECHOMINT	CCSM3MINT	ECHOMAXT	CCSM3MAXT	CCSM3P	ECHOP
Denit	−0.957	0.957	−0.957	0.954	−0.942	−0.683	−0.646	−0.639	−0.42	−0.172	0.128
	2.00E-07	2.00E-07	2.00E-07	2.00E-07	2.00E-07	2.00E-07	2.00E-07	3.32E-09	8.14E-04	1.84E-01	3.23E-01

	CO2	WETRN	WETON	DRYRN	DRYON	ECHOMINT	CCSM3MINT	ECHOMAXT	CCSM3MAXT	CCSM3P	ECHOP
N-uptake	−0.955	−0.953	−0.953	−0.953	0.95	−0.67	−0.664	−0.592	−0.45	−0.179	0.0715
	2.00E-07	2.00E-07	2.00E-07	2.00E-07	2.00E-07	2.00E-07	2.00E-07	5.63E-07	3.06E-04	1.68E-01	5.83E-01

Org-N – Organic nitrogen, NO_3_–inorganic nitrogen (nitrate), Denit– denitrification, N-uptake – plant nitrogen uptake, WETRN – Wet reduced nitrogen, WETON – Wet oxidized nitrogen, DRYON – Dry oxidized nitrogen, DRYRN – Dry reduced nitrogen, CO2–carbon dioxide, ECHOMINT– ECHO Minimum temperature, ECHOMAXT – ECHO maximum temperature, CCSM3MINT – CCSM3 minimum temperature, CCSM3MAXT – CCSM3 maximum temperature, ECHOP – ECHO precipitation, CCSM3P- CCSM3 precipitation.

**Table 4:** Spearman rank correlations for Nahunta. Spearman rho (ρ) values are on top and p-values are below. Terms are color-coded to help with interpretation. Atmospheric deposition terms are in the yellow to red colors, temperature is in blue colors, precipitation is in green colors and CO_2_ is white. Land cover change was not included in this correlation analysis because there are only five urban change percentage values from 2010 to 2070 (see Gabriel et al., 2016).

	ECHOP	CCSM3P	CCSM3MINT	DRYON	WETRN	WETON	DRYRN	CCSM3MAXT	CO2	ECHOMINT	ECHOMAXT
Org-N	0.651	0.589	0.508	−0.418	0.41	−0.41	−0.41	0.407	0.395	0.268	0.263
	2.00E-07	6.63E-07	3.42E-05	8.68E-04	1.10E-03	1.10E-03	1.10E-03	1.22E-03	1.74E-03	3.72E-02	4.11E-02

	DRYON	WETRN	WETON	DRYRN	CO2	CCSM3P	ECHOMINT	ECHOMAXT	ECHOP	CCSM3MAXT	CCSM3MINT
NO_3_	0.5	−0.475	−0.475	−0.475	−0.451	0.437	−0.371	−0.306	0.285	−0.187	−0.185
	4.68E-05	1.25E-04	1.25E-04	1.25E-04	2.96E-04	4.66E-04	3.35E-03	1.66E-02	2.60E-02	1.50E-01	1.54E-01

	DRYON	WETRN	WETON	DRYRN	CO2	ECHOMINT	CCSM3MAXT	CCSM3MINT	ECHOMAXT	ECHOP	CCSM3P
Denit	0.584	−0.564	−0.564	−0.564	−0.522	−0.463	−0.422	−0.373	−0.315	0.169	0.099
	8.96E-07	2.63E-06	2.63E-06	2.63E-06	1.91E-05	1.96E-04	7.69E-04	3.20E-03	1.37E-02	1.91E-01	4.46E-01

	DRYON	WETRN	WETON	DRYRN	CO2	ECHOMINT	CCSM3MINT	ECHOMAXT	CCSM3MAXT	CCSM3P	ECHOP
N-uptake	0.943	−0.94	−0.94	−0.94	−0.927	−0.641	−0.636	−0.605	−0.452	−0.228	−0.0585
	2.00E-07	2.00E-07	2.00E-07	2.00E-07	2.00E-07	6.28E-10	8.91E-09	2.41E-07	2.87E-04	7.71E-02	6.53E-01

Org-N – Organic nitrogen, NO_3_–inorganic nitrogen (nitrate), Denit– denitrification, N-uptake –plant nitrogen uptake, WETRN – Wet reduced nitrogen, WETON– Wet oxidized nitrogen, DRYON – Dry oxidized nitrogen, DRYRN – Dry reduced nitrogen, CO2–carbon dioxide, ECHOMINT- ECHO Minimum temperature, ECHOMAXT – ECHO maximum temperature, CCSM3MINT – CCSM3 minimum temperature, CCSM3MAXT – CCSM3 maximum temperature, ECHOP– ECHO precipitation, CCSM3P- CCSM3 precipitation.
