# Supplementary material for: Modeling the combined effects of changing land cover, climate, and atmospheric deposition on nitrogen transport in the Neuse River Basin
Source: J Hydrol Reg Stud. Author manuscript; Available in PMC 2019 Aug 1. (PMC6145828; doi:10.1016/j.ejrh.2018.05.004)
Supplement: supp [file NIHMS982016-supplement-supp.docx]

**Supporting Information Section**

**1.0 SWAT Simulation and Nitrogen Cycle**

In SWAT, watersheds are separated into sub-basins and “hydrologic response units” (HRUs). HRUs are land surface areas that contain the same combination of soil, land cover and slope class (Neitsch et al., 2005). SWAT is a semi-empirical, semi-distributed model which can be operated through an ArcGIS interface (ArcSWAT version 2009.93.5). The main components of SWAT are: climate, hydrology, land cover/plant growth, erosion, nutrients, pesticides, management and channel routing. SWAT was designed to determine impacts of climate and land management on water supply and water quality (Krysanova and Arnold, 2008). The SRS runoff curve number is used to predict runoff and infiltration. The runoff curve number method is a function of soil permeability, land cover and antecedent soil water conditions. SWAT performs calculations on a daily time step and we extracted results on a yearly basis.

SWAT simulates nitrogen cycles in the soil profile and groundwater (Neitsch *et al*., 2005). Within both media, nitrogen is highly reactive and exists in many forms. Nitrogen may be added to soil through fertilizer and manure application, atmospheric deposition (dry and wet) and biological fixation (Lam *et al*., 2011). Nitrogen may be removed from soil through plant uptake, soil erosion, leaching, volatilization, denitrification and runoff (Lam *et al*., 2011). In SWAT, there are five different pools of nitrogen in the soil: two pools are inorganic forms (ammonium [NH_4_^+^] and nitrate [NO^-^_3_]), while the other three are organic (fresh, active, and stable). Fresh organic nitrogen is associated with crop residue and microbial biomass, and the active and stable organic nitrogen pools are associated with soil humus. The fresh component is much more bioavailable than humus. Organic nitrogen associated with humus is partitioned into two pools (active, stable) to account for variations in availability of humic substances to mineralization. Humus is a complex mixture of organic substances that have been significantly modified from their original form over time, and contains other substances that have been synthesized by soil organisms (Lam *et al*., 2011). Typically, humus represents the majority of total soil organic matter and plays a major role in the ability of a soil to retain nutrients and water. Inorganic nitrogen may be transported with runoff, lateral flow or soil percolation. Inorganic nitrogen entering shallow groundwater in recharge from soil percolation may remain in groundwater, move into the main channel, or move into the soil zone in response to water deficiencies (Lam *et al*., 2011). Inorganic nitrogen may also move from the shallow soils to deep groundwater. For low-lying areas, groundwater flow is the primary transport pathway and plays an important role in the delivery of inorganic nitrogen to the main channel or to the soil zone.

Table S1 details SWAT calibration and validation results. As is commonly observed, results for nitrate calibration are weaker than flow, which may be due to several inter-related issues stemming from uncertainties or inaccuracies in observed data to misrepresentation of actual agricultural/land cover practices. The percent biases are within the acceptable range of ±75 % for nutrient calibration (Moriasi *et al.* 2007; Bosch, 2008; Silling and Wolter, 2009; Douglas-Mankin *et al. 2010*).

**Table S1** Calibration and validation results on flow (m^3^/s, monthly average) and nitrate load (kg/month) (results published in Gabriel et al. 2014a)

|  | **Little River** | | | | | | **Nahunta** | | | | | |
| --- | --- | --- | --- | --- | --- | --- | --- | --- | --- | --- | --- | --- |
|  | **Calibration** | | | **Validation** | | | **Calibration** | | | **Validation** | | |
|  | NS^*^ | r^2^ | PBIAS^†^ (%) | NS^*^ | r^2^ | PBIAS^†^% | NS^*^ | r^2^ | PBIAS^†^% | NS^*^ | r^2^ | PBIAS^†^% |
| Flow | 0.77 | 0.77 | -11.5 | 0.70 | 0.72 | 18.49 | 0.79 | 0.80 | -11.3 | 0.40 | 0.56 | 7.78 |
| Nitrate load | 0.24 | 0.35 | -35.3 | 0.47 | 0.54 | -16.8 | 0.15 | 0.32 | 21.6 | 0.03 | 0.26 | -26.0 |

*Nash-Sutcliffe Efficiency test

†Percent bias

**2.0 SWAT Model Input Data**

**2.1 Data Input and Processing**

The Soil Survey Geographic Database (SSURGO) (National Resources Conservation Service [NRCS]; <http://soils.usda.gov/survey/geography/ssurgo/>) was the source of soil input for the studied watersheds. SSURGO soil data was available for all necessary counties (Wayne, Johnson, Greene, Durham, Orange, and Person) within and surrounding both watersheds. These data represent surveys and compilations between 1990 and 2009. A digital elevation model (DEM) (<http://www.mapmart.com/Products/DigitalElevationModel/USGSNED.aspx>) at 30 m resolution was used to represent surface elevation within each watershed. For the Nahunta watershed, the 2009 Crop Data Layer (CDL: <http://datagateway.nrcs.usda.gov>) and 2001 National Land Cover Data Layer (NLCD: http://[www.epa.gov/mrlc/nlcd-2001.html](http://www.epa.gov/mrlc/nlcd-2001.html)) were used for land cover characterizations. For the Little River watershed, the 2009 CDL and 2006 NLCD (http://[www.mrlc.gov/nlcd06_data.php](http://www.mrlc.gov/nlcd06_data.php)) were used. The 2006 NLCD was used because we suspect the Little River watershed experienced clear-cutting for an area approximately 0.6 km^2^ near the watershed outlet that was not captured in the 2001 NLCD. Using the EPA’s landscape characterization program (http://[www.maps6.epa.gov](http://www.maps6.epa.gov)) the phenological record shows a sharp drop in normalized difference vegetation index (NDVI) after 2006 near the watershed outlet and a gradual increase in NDVI which likely indicates clear cutting (R. Lunetta pers. comm.). Therefore, to help account for this disturbance the 2006 NLCD was used instead of the 2001. The following HRU threshold limit delineation scheme was applied: 5% land use, 10% soil, 5% slope. Under this scheme, HRUs were not developed from land uses (land covers), soil types or surface slopes that were less than these percentages compared to the total areas of each. This scheme was developed to negate the consideration of numerous small HRUs that, if considered, would have negligible impacts on flow and nitrogen transport.

**2.2 Observed Flow and Nitrate Data**

Stream flow (m^3^/s) and nitrate (NO^-^_3_-N, mg/L) data were retrieved for both watersheds from 1990 to 2009. Watershed flow for the Little River watershed was retrieved at US Geological Survey (USGS) gauging station 208521324: Little River at SR1461 near Orange Factory, NC. Flow for the Nahunta watershed was retrieved at the USGS gauging station 2091000: Nahunta Swamp near Shine, NC. The USGS, National Weather Information Service (NWIS) (<http://waterdata.usgs.gov/nwis>) was the source of all surface flow data. Observed flow data was calculated on an average monthly basis from daily data. Observed nitrate data was retrieved from USEPA STORET (Storage and Retrieval) (<http://www.epa.gov/STORET/>). The frequency of collection for the nitrate concentrations was approximately monthly to bimonthly. Missing values for daily nitrate loads were determined using the USGS Estimator protocol (Richards *et al*., 1998). For information on field data retrieval and analytical quality assurance/control (QAQC) measures for the NWIS flow and USEPA STORET data, refer to the respective web links.

**2.3 Atmospheric Deposition of Nitrogen**

In this study we were specifically interested in evaluating temporal changes in nitrogen discharge as a function of land cover and climate changes; therefore, we used a constant atmospheric nitrogen deposition rate for each year from 2005 to 2070. This rate was the same for both watersheds. Nitrogen deposition rates were obtained from the USEPA’s Community Multi-scale Air Quality (CMAQ) Version 4.6 modeling system (Byun and Schere, 2006; USEPA 2012), applied as follows: 0.97 kg/ha-yr (dry reduced nitrogen), 16.5 kg/ha-yr (dry oxidized nitrogen), 1.62 kg/ha-yr (wet reduced nitrogen) (avg. 0.15 mg/L) and 7.00 kg/ha-yr (wet oxidized nitrogen) (avg. 0.66 mg/L). These rates reflect the “with” Clean Air Act Amendment (CAAA) scenario for year 2010. The CMAQ “with” CAAA scenario represents expected CAAA emissions measures implemented since 1990 to comply with rules promulgated through September 2005. For details on the CMAQ model and the “with” CAAA scenario, refer to *Benefits and Costs of the Clean Air Act from 1990 to 2020* (USEPA, 2011).

**2.4 Agricultural Land Practice and Soil Geochemical Information**

Several sources of information were used to represent actual agricultural practices and soil nitrogen levels for the studied watersheds (Table S2). For agriculture, the modifications involved crop rotations and nitrogen applications; in both watersheds, corn-soybean rotations were applied for both corn and soybean crop layers. Both watersheds contained varying numbers of concentrated feeding operations (CAFOs), poultry and swine operations which were represented through published values for fertilizer and manure applications on crop land (Ruddy et al. 2006). Specifically, two different fertilizer/manure application schemes were applied -- one for urban land uses and one for cropland/agricultural lands. Fertilizer and manure application rates varied per year for each watershed. Fertilizer application for urban land uses was evenly delivered as urea (CO (NH_2_)_2_), and ranged from 0.07 to 1.37 kg/ha-yr. Agricultural land received nitrogen application through fresh swine manure application and ranged from 8.2 to 8.93 kg/ha-yr. Fertilizer application to agricultural land uses was as elemental nitrogen, ranging form 5.50 to 38.0 kg/ha-yr. Overall, more nitrogen was applied to the Nahunta agricultural land uses. In all cases, fertilizer application decreased from the first year (1998) to the last year (2009) as estimated from (Ruddy *et al*., 2006). Crop turn-over for all agricultural layers was managed through heat units. Soil geochemistry data was obtained from published values for organic and inorganic nitrogen concentrations for the top three soil layers (Table S2).

**3.0 Parameter Sensitivity Analysis**

Following data collection and SWAT initialization, we performed parameter sensitivity analysis. The most sensitive model input parameters for flow and nutrient loading were identified with an automated sensitivity analysis procedure in SWAT. This sensitivity analysis was performed for flow and nutrient loading for the sub-watersheds that contained the outlet for the entire watershed. Latin Hypercube (LH) sampling and the one-factor-at-a-time (OAT) method was applied (Van Griensven *et al*., 2006) to determine parameter sensitivity for flow and nutrient loading. Ten LH intervals and an OAT parameter change value of 0.05 were applied. Under LH, samples were chosen across the full range of values (SWAT default) for each parameter assuming a uniform distribution. Following simulation, the overall effect of each parameter is ranked highest to lowest. The top 10^th^ percentile of all parameters for each category (flow, nutrients) was used for the next step, calibration and validation.

Table S3 shows calibrated parameter values for both watersheds as a result of calibration. The top 10^th^ percentile of input parameters identified in the parameter sensitivity analysis was used for calibration. In all, the input parameters found to be most sensitive, as indicated by those with p<0.001 in Table S3, closely agrees with past studies, in particular, Rouhani *et al*. (2009), Sexton *et al*. (2010), Lam *et al*. (2010),Wang *et al*. (2010) and Masih *et al*. (2011). Several other input parameters were used to fine tune the calibration: Gw_Delay.gw, Gw_Revap.gw, Surlag.bsn, AI1.wwq, Sol_No3.chm, Sol_Orgn.chm, Shallst_N.gw, Biomix.mgt, Sdnco.bsn, Cdn.bsn and Rsdco.bsn (see Table S2 for a description of each parameter).

**Table S2** Source information for land cover and soil data input categories

| **Category** | **Information Sources** |
| --- | --- |
| Fertilizer and manure applications | -NCDAC (2011)  -NASS (2011)  -NCSU (2011)  -Ruddy *et al*. (2006) |
| Soil geochemical properties | -NRCS (2011)  -Ibendahl and Fleming (2007)  -Christensen and MacAller (1985)  -Roelle and Aneja (2002) |
| Surface and groundwater chemistry | -NCDENR (2011)  -NCSU (2011)  -USGS NWIS (2011)  -USEPA STORET (2011) |
| Agricultural management practices (e.g. soil tillage, contouring, filter strip and tile drain practices) | -NC Cooperative Extension (2011)  -NC Department of Agriculture (2011)  -Folle *et al*. (2007) |
| Locations and characteristics for confined feeding operations (CAFOs), swine and poultry operations | -Spruill *et al*. (2005)  -NC One map (2011) |
| Surface flow and precipitation | -USGS NWIS (2011) |
| Atmospheric nitrogen deposition | -USEPA CASTNET (2011)  -NADP (2011) |

**Table S3** Input parameter values for flow and nitrate loading: Under Parameter Name, italicized input parameters were only used for nitrate load calibration. Non-italicized input parameters were only used for flow calibration

| **Parameter Description** | **Parameter Name** | **Mode of Change During Calibration^#^** | **Little River** | | **Nahunta** | | **Parameter Sensitivity Indicated by the SUFI-2 p-value** | |
| --- | --- | --- | --- | --- | --- | --- | --- | --- |
|  |  |  | **Un-calibrated value** | **Final calibrated value** | **Un-calibrated value** | **Final calibrated value** | **Little River** | **Nahunta** |
| SCS runoff curve number for moisture condition II | CN2.mgt* | r | 55.0-84.0 | 63.8-98.6 | 31-92 | 31.1-92.9 | 0.6162 | <0.0001 |
| Baseflow alpha factor | ALPHA_BF.gw | v | 0.048 | 0.19 | 0.048 | 0.95 | 0.9072 | 0.7378 |
| Manning's “ n” value for main channel | CH_N2.rte | v | 0.014 | 0.19 | 0.014 | 0.094 | 0.9486 | 0.3498 |
| Surface runoff lag coefficient | SURLAG.bsn | v | 4.0 | 4.51 | 4.0 | 8.75 | 0.7552 | 0.8346 |
| Soil evaporation compensation factor | ESCO.hru | v | 0.95 | 0.89 | 0.95 | 0.96 | 0.0363 | <0.0001 |
| Threshold depth of water in the shallow aquifer required for return flow to occur | GWQMN.gw | v | 0 | 69.1 | 0 | 254.2 | <0.0001 | <0.0001 |
| Maximum canopy storage | CANMX.hru | v | 0 | 92.6 | 0 | 45.2 | 0.5392 | <0.0001 |
| Available water capacity of soil layer | SOL_AWC1.sol* | r | 0.11-0.2 | 0.134-0.244 | 0.07-0.7 | Not changed | 0.1994 | - |
| Groundwater “revap” coefficient | GW_REVAP.gw | v | 0.02 | 0.10 | 0.02 | Not changed | 0.8954 | - |
| Threshold depth of water the shallow aquifer for “revap” or percolation to the deep aquifer to occur | REVAPMN.gw | v | 1 | 403.3 | 1 | Not changed | 0.0011 | - |
| Groundwater delay time | GW_DELAY.gw | v | 31 | 60.7 | 31 | Not changed | <0.0001 | - |
| Deep aquifer percolation fraction | RCHRG_DP.gw | v | 0.05 | 0.072 | 0.05 | 0.028 | <0.0001 | <0.0001 |
| Fraction of algal biomass that is nitrogen | *AI1.wwq* | v | 0.08 | 0.072 | 0.08 | 0.087 | 0.5749 | 0.4346 |
| Nitrate percolation coefficient | *NPERCO.bsn* | v | 0.2 | 0.50 | 0.2 | 0.10 | 0.6572 | <0.0001 |
| Phosphorous percolation coefficient | *PPERCO.bsn* | v | 10 | 16.0 | 10 | 13.43 | 0.0433 | 0.9713 |
| Initial nitrate concentrations in soil layers one, two an three | *SOL_NO3.chm* | v | 6.25†. 3.0†, 1.5† | 0.01, 0.01,0.01 | 13.46†, 6.73†, 3.3† | 0.264, 6.73, 3.3 | 0.3979 | 0.0003 |
| Initial organic nitrogen concentrations in soil layer one, two and three | *SOL_ORGN.chm* | v | 25.0†, 12.5†, 6.25† | 10.43, 12.5, 6.25 | 50.0†, 25.0†, 12.5† | 19.19, 25.0, 12.5 | 0.9965 | 0.0540 |
| Nitrate concentration in shallow aquifer | *SHALLST_N.gw* | v | 1.5† | 178.3 | 7.75† | 419.9 | 0.6735 | 0.3652 |
| Biological mixing efficiency | *BIOMIX.mgt* | v | 0.20 | 0.03 | 0.20 | 0.655 | 0.1491 | 0.5470 |
| Average slope length | *SLSUBBSN.hru** | r | 60.9-121.9 | Not changed | 60.8-121.8 | 59.7-119.5 | - | 0.4190 |
| Residue decomposition coefficient | *RSDCO.bsn* | v | 0.05 | 0.03 | 0.05 | Not changed | 0.7458 | - |
| Denitrification threshold water content | *SDNCO.bsn* | v | 1 | 0.80 | 1 | 0.99 | No data‡ | No data‡ |
| Denitrification exponential rate coefficient | *CDN.bsn* | v | 1.4 | 0.10 | 1.4 | 0.01 | No data‡ | No data‡ |
| Phosphorus soil partitioning coefficient | *PHOSKD.bsn* | v | 175 | 152.1 | 175 | Not changed | 0.9675 | - |

*varies by watershed/HRU; values shown are the minimum and maximum value

†values determined from literature review

‡ No sensitivity data is available as manual calibration was used, # “r” refers to application by multiplication and “v” refers to application by replacement
